# Diagnostic performance of circulating MicroRNAs in acute ischemic stroke: A protocol for systematic review and meta-analysis

**DOI:** 10.1097/MD.0000000000022353

**Published:** 2020-10-02

**Authors:** Wenzhai Cao, Ting Zhang, Lizhen Wang, Jing Fu, Hongchuan Jin

**Affiliations:** a Laboratory of Cancer Biology, Key Lab of Biotherapy in Zhejiang, Sir Run Run Shaw Hospital, Medical School of Zhejiang University, Hangzhou, Zhejiang; b The Institute of Cardiovascular Research, SouthWest Medical University, Luzhou, Sichuan; c Department of Nursing, Sichuan Vocational College of Health and Rehabilitation, Zigong; d School of Clinical Medicine, Chengdu University of Traditional Chinese Medicine, Chengdu; e School of Nursing, SouthWest Medical University, Luzhou, Sichuan, P.R. China.

**Keywords:** biomarker, diagnosis, ischemic stroke, meta-analysis, microRNA

## Abstract

**Introduction:**

Increasing evidences showed differential expression of circulating microRNAs (miRNAs) in patients with acute ischemic stroke (AIS), indicating that miRNAs might serve as promising biomakers in the diagnosis of AIS. However, their accuracy has not been systematically evaluated, so it is necessary to conducted a meta-analysis to evaluate the diagnostic value of miRNAs in AIS patients.

**Methods:**

PubMed, EMBASE, Cochrane Library, Web of Science, Medline, China National Knowledge Infrastructure (CNKI) will be searched for the relevant studies that explored the potential diagnostic values of miRNAs in AIS patients from inception to August 2020. Data will be extracted by two researchers independently; risk of bias of the meta-analysis will be evaluated by the Quality Assessment of Diagnostic Accuracy Studies-2 (QUADAS-2). Data will be synthesised and heterogeneity will be evaluated. All of the above statistical analysis will be performed using Stata V.15.0 and Meta-disc V.1.4.

**Results:**

This study will assess the pooled diagnostic performance of circulating miRNAs in AIS.

**Conclusion:**

This study will clarify confusions about the specificity and sensitivity of circulating miRNAs in diagnosing AIS, which could further guide the promotion and application of them.

Open Science Framework (OSF) registration number: 2020, August 19. https://osf.io/6tjf3.

## 1 Introduction

Stroke, a most devastating diseases, is the second cause of death worldwide and the leading cause of disability in adults.[^1^,^2^] Broadly, stroke is divided into ischemic and hemorrhagic categories, with ischemic stroke accounting for ∼87% of the total stroke cases.^[1]^ The most promising treatment of ischemic stroke are thrombolysis and thrombectomy, but the strict time window is required for those therapies.^[3]^ Nowadays, diagnosis of acute ischemic stroke (AIS) principally relies on advanced neuroimaging techniques including computed tomography (CT) and magnetic resonance imaging (MRI), whereas there are inherent limitations. CT is not sufficiently sensitive to super-early stage of cerebral infarction, as 40% to 50% of all AIS events lack abnormalities on admission CT scans.^[4]^ MRI has some obstacles in application, such as the limited availability,^[5]^ high cost of the scanners, and substantial time for the procedure.^[6]^ Therefore, a more rapid and simple tool is essential for prompt diagnosis of AIS.

MicroRNAs (miRNAs), single-stranded non-coding RNAs, which could be released into the circulation, hold promise for diagnostic biomarkers due to their easy detection, stability in blood and cell-type specific expression patterns.^[7]^ miiRNAs have been reported to play several possible underlying mechanisms in the occurrence and development of stroke, including cellular apoptosis, neuroinflammation, and oxidative stress.[^8^,^9^] Previous studies have found abnormal expression of miRNAs in patients with AIS, which suggests potential diagnostic value of miRNAs.[^10^,^11^] Nonetheless, the inadequate sample size, inconsistent subjects or diverse detection techniques lead to considerable discrepancy among those studies and inconsistent results. In this study, we will evaluate the current literature focusing on the association between the circulating miRNAs and AIS by meta-analysis.

## 2 Methods

### 2.1 Study registration

The protocol of the systematic review has been registered. Registration: OSF Preregisration. 2020, August 19. https://osf.io/6tjf3. It has been reported following the guideline of Preferred Reporting Items for Systematic Reviews and MetaAnalysis Protocol statement.^[12]^

### 2.2 Inclusion criteria for study selection

#### 2.2.1 Type of studies

This review will include cohort, case-controlled studies that evaluate the value of miRNAs in the diagnosis of AIS.

#### 2.2.2 Type of participants

All the patients who were diagnosed AIS based on neuroimaging (MRI/CT) will be included in this review, regardless gender, age, population, and severity of AIS.

#### 2.2.3 Type of index test

Index test: circulating miRNAs were used in detecting patients with AIS. However, we will exclude case reports, reviews, cell, or animal studies.

#### 2.2.4 Outcome measurements

Outcomes are the Pooled SEN, SPE, PLR, NLR, DOR, AUC, and their 95%*CI*.

### 2.3 Data sources and search strategy

This study will perform a literature search in PubMed, EMBASE, Cochrane Library, Medline and Web of Science, and CNKI. We will limit our search in the English and Chinese language and make a final search on August 20, 2020. The search strategy of Medline was shown in Table 1. Other electronic databases will be used the similar retrieval strategies.

**Table 1:**
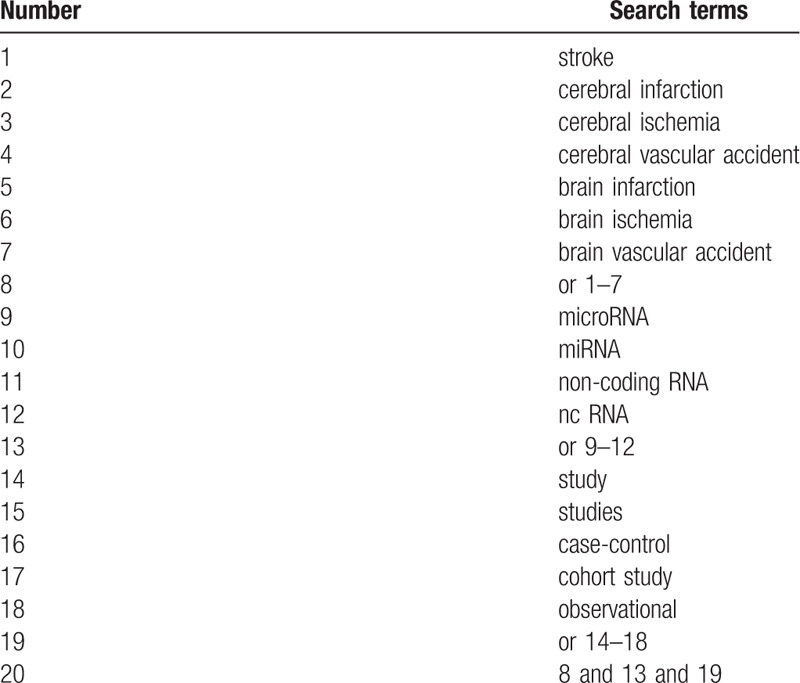
Search strategy applied in MEDLINE database.

### 2.4 Data collection and analysis

#### 2.4.1 Study selection

Two reviewers will screen the titles and abstracts independently. Then, the full text of potential studies will be retrieved for further selection according to the inclusion criteria. Any disagreements will be resolved by a third researcher.

#### 2.4.2 Data extraction

Two reviewers will independently extract the data using a standardized form and confirm by a third researcher. If there are missing or unclear information, we will contact the authors to confirm them. The data extraction form will include the following items: first author, publication year, regions, sample size, sample types, control group, studied miRNAs, RNA detection methods, data needed for diagnostic meta-analysis (sensibility and specificity data).

### 2.5 Quality assessment

The methodological quality of the included studies will be assessed using Quality Assessment of Diagnostic Accuracy Studies-2 (QUADAS-2) criteria in RevMan 5.3 software.^[13]^ The tool consists of 4 key domains: patient selection, index test, reference standard and flow and timing, evaluated the risk of bias and concerns about clinic applicability of all included publications. Two reviewers will independently and blindly evaluate the studies. Any discrepancies between the two reviewers will be resolved by consensus.

### 2.6 Statistical analysis

We will calculate the pooled SEN, SPE, PLR, NLR, DOR, and their 95% CI. Besides, the pooled diagnostic value of miRNAs through the SROC and AUC will be tested. The Spearman correlation coefficient between the logit of sensitivity and logit of 1-specificity will be calculated to evaluate the threshold effect, and a *P* value < .05 shows significant threshold effect. Heterogeneity caused by non-threshold effect will be assessed by means of the Cochran *Q* test and the inconsistency index (*I* ^2^) measurement.^[14]^ Heterogeneity will be deemed significant with *P* < .1 or *I* ^2^ > 50%, and a random-effects model will be applied. All of the above statistical analysis will be performed using Stata V.15.0 and meta-disc V.1.4. *P* values < .05 will be considered statistically significant.

### 2.7 Subgroup analysis

In order to further investigation of potential heterogeneity, subgroup analyses will be performed based on population, miRNA type, characteristics of control, and sample type.

### 2.8 Sensitivity analysis

This review will perform sensitivity analysis to test the stability of study findings. If some studies substantially change the pooled RR in the result of meta-analysis, they will be removed.

### 2.9 Reporting bias

Detection of publication bias will be tested by funnel plots and associated regression tests.[^15^,^16^]

### 2.10 Ethics and dissemination

This review will extract data from published studies, so examination and agreement by the ethics committee are not required in this study. It will be published in a relevant peer reviewed journal.

## 3 Discussion

Nowadays, as the clinical examination may be non-decisive and neuroimaging could not be feasible on some occasions, non-invasive blood biomarkers are imperative for AIS. However, no universally acknowledged biomarkers could be available for routine application in the setting of diagnosis, differentiation, or risk stratification for acute stroke.^[17]^ miRNAs, abundantly expressed in the brain, involved in a variety of physiological and pathological cellular processes and acted as prospective biomarkers that reflect body status.^[18]^ Although many studies had indicated abnormal miRNA expression in patients with AIS, a systematic review and meta-analysis were warranted to compile and synthesize the available data and thus address some of the questions.

This is the first meta-analysis to comprehensively search and summarize the evidence on the effect of circulating miRNAs in diagnosis of AIS. The results of this review will provide clinical evidence and represent a possibility and future direction of AIS diagnosis.

## Author contributions

**Conceptualization:** Jing Fu, Hongchuan Jin.

**Data curation:** Wenzhai Cao, Ting Zhang, Lizhen Wang.

**Software:** Wenzhai Cao, Ting Zhang.

**Writing – original draft:** Wenzhai Cao.

**Writing – review & editing:** Ting Zhang.
